# Enhancing YOLOv11 with Large Kernel Attention and Multi-Scale Fusion for Accurate Small and Multi-Lesion Bone Tumor Detection in Radiographs

**DOI:** 10.3390/diagnostics15161988

**Published:** 2025-08-08

**Authors:** Sihan Chen, Youcheng Peng, Yingxuan Liu, Pengyu Wang, Tao Liu

**Affiliations:** 1 Sydney Smart Technology College, Northeastern University at Qinhuangdao, Qinhuangdao 066004, China; 202219164@stu.neuq.edu.cn (S.C.); 202219285@stu.neuq.edu.cn (Y.P.); 2 School of Control Engineering, Northeastern University at Qinhuangdao, Qinhuangdao 066004, China; 1001287@neuq.edu.cn; 3 School of Resources and Materials, Northeastern University at Qinhuangdao, Qinhuangdao 066004, China; 202216175@stu.neuq.edu.cn; 4 School of Mathematics and Statistics, Northeastern University at Qinhuangdao, Qinhuangdao 066004, China

**Keywords:** YOLOv11, primary bone tumors, X-ray radiography, small-object detection

## Abstract

**Objectives:** Primary bone tumors such as osteosarcoma and chondrosarcoma are rare but aggressive malignancies that require early and accurate diagnosis. Although X-ray radiography is a widely accessible imaging modality, detecting small or multi lesions remains challenging. Existing deep learning models are often trained on small, single-center datasets and lack generalizability, limiting their clinical effectiveness. **Methods:** We propose the YOLOv11-MTB, a novel enhancement to YOLOv11 integrating multi-scale Transformer-based attention, boundary-aware feature fusion, and receptive field augmentation to improve detection of small and multi-focal lesions. The model is trained and evaluated on two multi-center datasets, including the BTXRD dataset containing annotated radiographs with lesion types and bounding boxes. **Results:** YOLOv11-MTB achieves state-of-the-art performance on bone tumor detection tasks. It attains a mean average precision (mAP) of 79.6% on the BTXRD dataset, outperforming existing methods. In clinically relevant categories, the model achieves small-lesion mAP of 55.8% and multi-lesion mAP of 63.2%. **Conclusions:** The proposed YOLOv11-MTB framework demonstrates promising generalization and accuracy for primary bone tumor detection in radiographic images. Its performance in detecting small and multiple lesions suggests potential for clinical application.

## 1. Introduction

Primary bone tumors, such as osteosarcoma and chondrosarcoma, are rare but highly aggressive malignancies that often require early diagnosis for effective clinical intervention [1]. X-ray radiography is the first-line imaging modality due to its availability and diagnostic value in assessing lesion morphology, destruction patterns, and periosteal reactions [2,3,4]. However, because of the low incidence and clinical rarity of primary bone tumors, many radiologists lack sufficient diagnostic experience, which may lead to misdiagnosis or delayed treatment [5].

Prior studies on computer-aided diagnosis (CAD) of bone tumors primarily focus on handcrafted feature extraction and classical machine learning classifiers [6,7]. With the rise of convolutional neural networks (CNNs), end-to-end deep learning approaches have gained traction, such as ResNet-based classifiers for bone lesion identification [8] and UNet variants for segmentation [9]. More recently, object detection frameworks including Faster R-CNN and RetinaNet have been adapted to localize tumors in musculoskeletal radiographs [10,11]. However, these models often suffer from limited spatial resolution and weak detection performance on small lesions. Transformer-based models have also been explored in medical imaging [12,13], but their application in bone radiography remains limited. Compared to these methods, YOLOv11 offers a more balanced tradeoff between detection accuracy and computational efficiency, making it a promising candidate for real-time clinical deployment.

In recent years, deep learning (DL) models have demonstrated significant potential in medical imaging tasks such as classification, detection, and segmentation [14,15]. Several studies have explored the use of DL in detecting bone tumors from radiographs [16,17,18]; however, these efforts are constrained by limited public datasets, weak annotations, or an inability to generalize across institutions. Most existing models are trained on single-center datasets, and many rely on manually cropped tumor regions, which limit their clinical applicability [19]. Furthermore, small and multi lesions present additional challenges to conventional detection models.

YOLO (You Only Look Once) models are widely used in real-time object detection due to their speed and accuracy. However, prior works in the medical imaging domain mostly use earlier versions such as YOLOv3–v5 [20,21,22], which are suboptimal for small object detection in complex radiographic backgrounds due to their limited receptive field and weaker feature extraction capabilities, particularly for small, spatially dispersed lesions. Earlier YOLO versions struggled to maintain high accuracy in such scenarios because they relied on fixed receptive fields and lacked effective multi-scale feature fusion, which is crucial for detecting small and multi-focal lesions in medical images. YOLOv11 [23], a recent advancement in the YOLO family, introduces enhancements such as the C3k2 module, C2fPSA attention, and spatial pyramid pooling fast (SPPF), which allow the model to capture a wider range of spatial contexts and improve multi-scale detection. These modifications make YOLOv11 effective for bone tumor detection, a domain where small lesion detection is a significant challenge. Nevertheless, its application to bone tumor detection has not been systematically studied, and the improvements introduced by YOLOv11 could offer advantages over previous versions in clinical settings.

To address these limitations, we propose a YOLOv11-based architecture, with novel modifications that incorporate large kernel attention, multiscale receptive field tuning, and feature-level fusion strategies specifically designed for small and multi-lesion detection in bone tumor radiographs. Our model is evaluated on two multi-center datasets, including the newly released BTXRD dataset [5], which contains 3746 X-ray images annotated with lesion type, anatomical site, imaging angle, bounding boxes, and segmentation masks, while the overall mean average precision (mAP) shows a modest improvement, our model achieves significant gains in clinically critical subcategories, especially for small-object and multi-object scenarios.

The main contributions of this paper are as follows:

- We propose an improved YOLOv11-based architecture for bone tumor detection and classification, addressing the clinical gap of small bone tumor detection. Small lesions, which are frequently missed in real-world datasets, exhibit a miss rate of 43% in the BTXRD dataset using YOLOv8. Our method, YOLOv11-MTB, reduces this miss rate to 22%, improving detection precision and increasing the overall mAP from 73.1 to 79.6.
- We perform extensive evaluation on two multi-center datasets, including the publicly available BTXRD dataset, verifying the generalizability and robustness of the proposed method across imaging centers and conditions.
- Our method achieves state-of-the-art results on both detection and classification tasks, particularly improving mAP for small-object and multi-lesion categories.
- We provide detailed ablation studies to illustrate how our architectural improvements contribute to the final performance, with implications for future clinical deployment.

## 2. Methods

YOLOv11 is a recent advancement in the YOLO object detection series that enhances both speed and accuracy through architectural innovation [23]. Released in September 2024, YOLOv11 underwent a series of structural upgrades aimed at improving computational efficiency without sacrificing detection precision. This makes it particularly suitable for medical imaging scenarios, where computational cost and model generalizability are critical.

The architecture of YOLOv11 introduces novel components such as C3k2 and C2fPSA modules, which jointly contribute to improved feature extraction and spatial reasoning while reducing the number of parameters compared to earlier YOLO versions.

C3k2 Module: The C3k2 block is a computationally efficient implementation of the cross-stage partial (CSP) bottleneck module. In comparison to conventional attention modules, such as the squeeze-and-excitation (SE) module and the convolutional block attention module (CBAM), the PSA mechanism in the C3k2 block focuses on spatial feature selection across different scales, enhancing efficiency by applying attention mechanisms more selectively, whereas SE and CBAM are typically applied uniformly across all channels or spatial locations. It replaces the standard C2f blocks in both the backbone and neck with a more efficient depthwise separable convolution design. Specifically, it employs a stack of three depthwise convolutions with progressively larger kernel sizes (k=13, k=17, k=21), which expands the receptive field and enables multi-scale context capture:

(1) $$y=\sum_{k\in \{13,17,21\}}{\mathrm{DWConv}}_{k}\left(x\right),$$

where ${\mathrm{DWConv}}_{k}$ denotes a depthwise convolution with kernel size *k* applied to the input feature *x*. This design ensures that small and spatially dispersed lesions in radiographs can be effectively aggregated and localized.

C2fPSA Module: The cross-stage partial parallel split attention (C2fPSA) module combines the structural advantages of CSP with an attention mechanism designed for spatial information selection. The PSA mechanism splits the feature maps into parallel branches, processes them independently, and fuses the outputs via attention-weighted summation:

(2) $$y=\sum_{i=1}^{n}\alpha_{i}f_{i}\left(x\right),\;\sum_{i}\alpha_{i}=1,$$

where $f_{i}\left(x\right)$ represents the *i*-th attention branch, and $\alpha_{i}$ denotes its corresponding learned attention weight. This allows the model to focus on diagnostically relevant regions, such as lesion boundaries, and suppress irrelevant background noise.

Backbone and Neck: The backbone employs layered C3k2 and C2fPSA modules for hierarchical feature encoding, followed by a neck composed of an spatial pyramid pooling fast (SPPF) module and a path aggregation network (PAN) structure for feature pyramid fusion. The SPPF enables efficient multi-scale spatial pooling, crucial for lesions of variable size and shape.

Decoupled Detection Head: The detection head in YOLOv11 is fully decoupled, with separate branches for classification and regression, enabling more specialized feature tuning. This architecture improves convergence and accuracy in multi-class, multi-instance detection scenarios like bone tumor analysis.

Anchor-Free Loss Function: YOLOv11 adopts an anchor-free detection strategy, simplifying the label assignment process. The overall loss function is a weighted sum of classification, bounding box regression, and objectness sub-losses:

(3) $$L_{\mathrm{total}}=\lambda_{cls}L_{cls}+\lambda_{box}L_{box}+\lambda_{obj}L_{obj},$$

where $\lambda_{cls}$, $\lambda_{box}$, and $\lambda_{obj}$ are task-specific hyperparameters balancing the learning objectives. $L_{cls}$ is the classification loss, $L_{box}$ denotes the bounding box regression loss, and $L_{obj}$ represents the objectness loss. This design reduces the hyperparameter tuning burden and increases detection robustness in medical domains.

### 2.1. Model Improvements for Bone Tumor Detection

To address the challenges of detecting small and multi-focal bone tumors in radiographs, we introduce a series of complementary enhancements to the YOLOv11 architecture, integrating medical domain knowledge with advanced computer vision techniques. These modifications focus on improving contextual understanding, multi-scale feature fusion, view-specific adaptation, and class-balanced learning.

**Figure 1:**
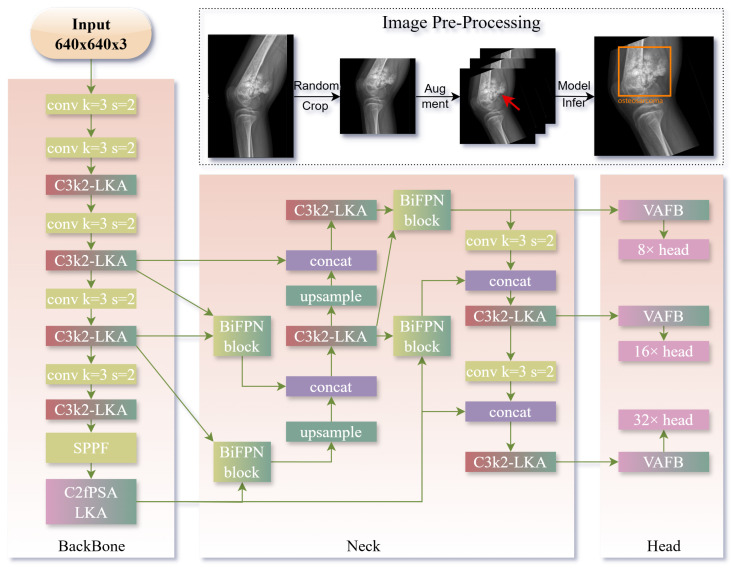
YOLOv11-MTB Framework: Overall Architecture and Data Preprocessing Pipeline (Top: Preprocessing workflow where raw images undergo random cropping and data augmentation to generate training inputs. The red arrow indicates the location of the lesion. Model outputs are visualized as orange bounding boxes with class labels and confidence scores. Main: Architecture diagram of YOLOv11-MTB, gradient-colored blocks highlight modules modified from YOLOv11).

The overall architecture of the YOLOv11-MTB framework is illustrated in Figure 1. The framework consists of four main components: (1) enhanced YOLOv11 with large kernel attention modules, (2) multi-scale feature fusion neck with BiFPN-inspired hierarchical aggregation, (3) angle-aware detection heads, and (4) adaptive focal loss for classification and localization.

Contextual Boundary Refinement with Large Kernel Attention: Bone tumors often exhibit blurred boundaries and subtle morphological features in X-ray images, necessitating enhanced contextual modeling. We introduce a large kernel attention (LKA) module to capture long-range anatomical dependencies while maintaining computational efficiency. Given an input feature map $X\in R^{C\times H\times W}$, the LKA module computes an attention map A using a depthwise dilated convolution with a large kernel size (k=31) and dilation factor d=2:

(4) $$A=\sigma \left({\mathrm{DWConv}}_{k=31,d=2}\left(X\right)\right)$$

where σ denotes the sigmoid activation function. The refined feature map $X^{\prime }$ is generated by element-wise multiplication of the attention map with the input features, followed by a residual connection:

(5) $$X^{\prime }=A⊙X+X$$

This design enables the network to focus on global structural cues while preserving local details, critical for delineating tumor boundaries in low-contrast radiographs. Group normalization GN(·) is applied after the residual operation to stabilize training across varying imaging conditions.

(6) $$\mathrm{GN}\left(X^{\prime }\right)=\frac{X^{\prime }-\mu }{\sqrt{\sigma^{2}+\epsilon }}\cdot \gamma +\beta$$

where μ and $\sigma^{2}$ are the mean and variance computed across groups, γ and β are learnable scale and shift parameters, and ϵ is a small constant for numerical stability. This normalization step ensures consistent feature scaling, which is crucial for effective attention learning in medical imaging tasks.

**Figure 2:**
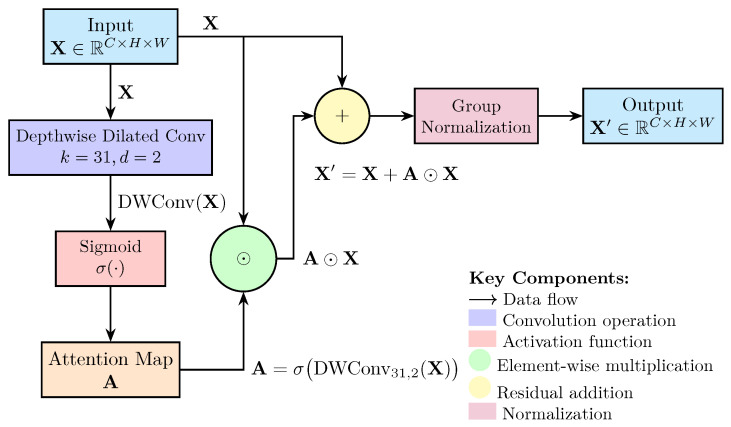
Architecture of the Large Kernel Attention Module. The module employs depthwise dilated convolution with kernel size 31 and dilation factor 2, followed by sigmoid activation and residual connection with group normalization.

The total number of parameters in the LKA module is significantly reduced compared to traditional attention mechanisms, making it suitable for real-time applications in clinical settings. The architecture of the LKA module is shown in Figure 2.

This component is strategically positioned within each C3k2 block in the backbone, as shown in Figure 1. Specifically, one LKA module is integrated after each C3k2 block to enhance contextual feature extraction at multiple scales throughout the feature hierarchy.

Hierarchical Multi-Scale Feature Fusion for Small Lesion Detection: Small bone tumors (often <32 pixels in diameter) are challenging to detect due to rapid downsampling in deep neural networks. To address this, we adopt a BiFPN-inspired hierarchical feature fusion strategy that dynamically aggregates features across multiple scales. This choice was made because BiFPN outperforms traditional methods like FPN and NAS-FPN in handling feature fusion across multiple scales. BiFPN’s learnable feature aggregation mechanism allows it to prioritize more informative features, which is particularly important for small lesion detection in radiographic images. For each output level *i*, the fused feature $F_{i}$ is computed as:

(7) $$F_{i}=\sum_{j\in N(i)}{\hat{w}}_{ij}\cdot U_{j},\;{\hat{w}}_{ij}=\frac{\exp(w_{ij})}{\sum_{k\in N(i)}\exp\left(w_{ik}\right)}$$

where N(i) denotes the set of adjacent feature levels, $U_{j}$ represents appropriately upsampled or downsampled features from level *j*, and $w_{ij}$ are learnable scalar weights. This weighted fusion allows the network to prioritize informative features from different scales, enhancing the detectability of small lesions. Depthwise separable convolutions and cross-level batch normalization are applied after each fusion step to refine features and maintain consistent scaling.

This enhancement is strategically positioned within the neck of our framework. Specifically, it is implemented in the BiFPN block shown in Figure 1, where multi-scale features from different backbone levels are hierarchically fused to enhance small lesion detection capabilities.

View-specific Feature Adaptation with Angle-aware Encoding Block (VAFB): X-ray images acquired from multiple viewpoints (e.g., frontal, lateral), each presenting distinct anatomical projections. To leverage this view information, we introduce angle-aware positional encoding to explicitly incorporate imaging metadata into the detection pipeline. A one-hot vector $a\in {\left\{0,1\right\}}^{K}$ (where *K* is the number of view types) is concatenated with the intermediate feature vector $f\in R^{D}$ and projected through a multi-layer perceptron (MLP):

(8) $$\hat{f}=\mathrm{MLP}\left(\left[f;a\right]\right)$$

An auxiliary view classification loss $L_{\mathrm{view}}$ is added to the training objective to encourage the network to learn view-dependent feature representations:

(9) $$L_{\mathrm{view}}=-\sum_{k=1}^{K}a_{k}\log\left({\hat{a}}_{k}\right)$$

This approach reduces view-specific false positives by enabling the model to adapt its feature interpretation based on the acquisition angle, improving generalization across diverse imaging protocols.

This component is strategically positioned within the detection head of our framework. Specifically, it is integrated into the view-aware feature block (VAFB) shown in Figure 1, where view-specific adaptations are applied before the final classification and localization predictions.

Class-balanced Learning with Adaptive Focal Loss: Addressing severe class imbalance between normal, benign, and malignant cases is critical for accurate diagnosis. We employ an adaptive focal loss framework that dynamically adjusts the focusing parameter γ and class weights $\alpha_{t}$ based on lesion characteristics:

(10) $$L_{\mathrm{focal}}=-\alpha_{t}{\left(1-p_{t}\right)}^{\gamma_{t}}\log\left(p_{t}\right)$$

where $p_{t}$ is the predicted probability for the true class *t*, $\alpha_{t}$ is a class-specific weight, and $\gamma_{t}$ is an adaptive focusing parameter. For small lesions (size < 32 × 32 pixels), $\gamma_{t}$ is increased to 2.5 to emphasize hard-to-detect instances, while standard focal loss (γ=2) is applied to larger lesions. Class weights $\alpha_{t}$ are set inversely proportional to class frequency, prioritizing rare malignant cases ($\alpha_{\mathrm{malignant}}=0.75$) over benign ($\alpha_{\mathrm{benign}}=0.5$) and normal ($\alpha_{\mathrm{normal}}=0.25$) samples.

Integrated System Architecture: These enhancements are seamlessly integrated into the YOLOv11 framework, forming a cohesive system optimized for bone tumor detection. The modified architecture processes input radiographs through (1) the enhanced backbone with LKA modules for contextual feature extraction, (2) the BiFPN neck for multi-scale fusion, (3) angle-aware detection heads for view-specific classification and localization, and (4) adaptive focal loss for balanced training. The complete loss function is formulated as:

(11) $$L_{\mathrm{total}}=L_{\mathrm{focal}}+\lambda_{\mathrm{loc}}L_{\mathrm{GIoU}}+\lambda_{\mathrm{view}}L_{\mathrm{view}}$$

where $L_{\mathrm{GIoU}}$ is the generalized intersection over union loss for bounding box regression, and $\lambda_{\mathrm{loc}}=1.0$, $\lambda_{\mathrm{view}}=0.1$ are hyperparameters balancing the contributions of each loss component.

### 2.2. Implementation Details

All models were implemented in PyTorch 2.1 and trained using four NVIDIA A100 GPUs. The input resolution was set to 640×640. In terms of runtime, the proposed model’s inference speed was evaluated during testing. It achieves an inference rate of approximately 97.85 frames per second (FPS), which corresponds to a latency of around 10.21 milliseconds per image. This performance allows for real-time processing, which is essential for deployment in clinical environments where rapid results are crucial. We used mixed-precision training to reduce memory overhead and increase training speed. The optimizer was AdamW with the following configuration:

- Initial learning rate: $5\times 10^{-4}$;
- Weight decay: $1\times 10^{-2}$;
- Scheduler: Cosine Annealing with warm-up for the first 5 epochs.

All experiments follow a consistent training configuration:

- Training duration: 300 maximum epochs;
- Evaluation: Metrics computed every 100 iterations;
- Early stopping: Activated if no improvement for 10 consecutive evaluations;
- Model selection: Final results reported using the best-performing weights.

This standardized setup ensures fair comparison between our proposed method and baseline approaches.

Data augmentation techniques included:

- Random crop;
- Resize to (640, 640);
- Horizontal flipping (*p* = 0.5);
- Random rotation in range $\left[-20^{\circ },20^{\circ }\right]$;
- Contrast-limited adaptive histogram equalization (CLAHE);
- Random brightness/contrast adjustments.

### 2.3. Evaluation Metrics

To quantitatively assess the performance of our method, we employed a series of evaluation metrics tailored for both the object detection and classification tasks.

For the object detection task, we utilized the mean average precision (mAP) across a range of Intersection over Union (IoU) thresholds from 0.5 to 0.95 in steps of 0.05, denoted as mAP. Formally, for a given IoU threshold τ, the average precision (AP) is defined as:

$${\mathrm{AP}}_{\tau }=\int_{0}^{1}p\left(r\right)\;dr,$$

where p(r) is the precision–recall curve as a function of recall *r*. The mean AP is then calculated as:

$$\mathrm{mAP}=\frac{1}{\left|T\right|}\sum_{\tau \in T}{\mathrm{AP}}_{\tau },\;T=\left\{0.5,0.55,\ldots ,0.95\right\}.$$

To specifically evaluate model sensitivity to small lesions—often indicative of early-stage tumors—we computed the mAP restricted to ground truth objects with area less than 32×32 pixels. Similarly, to assess robustness in complex scenarios, we computed multi-lesion mAP over the subset of test images containing three or more distinct lesions.

In addition to AP-based metrics, we employed the F1-score, which harmonizes precision and recall:

$$F1=\frac{2\cdot \mathrm{Precision}\cdot \mathrm{Recall}}{\mathrm{Precision}+\mathrm{Recall}},$$

where $\mathrm{Precision}=\frac{TP}{TP+FP}$ and $\mathrm{Recall}=\frac{TP}{TP+FN}$, with TP, FP, and FN representing the numbers of true positives, false positives, and false negatives, respectively. The precision–recall curve (PRC) was also plotted to visualize the trade-off across varying thresholds.

For the classification task, which involves binary discrimination between benign and malignant lesions, we adopted overall accuracy, computed as:

$$\mathrm{Accuracy}=\frac{TP+TN}{TP+TN+FP+FN},$$

to measure the proportion of correctly classified samples. To address potential class imbalance, we computed the macro-averaged F1-score:

$$F1_{\mathrm{macro}}=\frac{1}{C}\sum_{i=1}^{C}F1_{i},$$

where *C* is the number of classes (in our case, 2 or 3) and $F1_{i}$ is the F1-score for class *i*.

In addition, the standard deviation (SD) of the metric values is calculated to provide a measure of the variability or consistency of the model’s performance. The SD is defined as:

$$\mathrm{SD}=\sqrt{\frac{1}{N}\sum_{i=1}^{N}{\left(x_{i}-\mathrm{Mean}\right)}^{2}}$$

where $x_{i}$ represents the individual metric values, and the mean is the average of those values.

To demonstrate model robustness across classification thresholds, we employ the area under the precision–recall curve (AUC). This metric provides a comprehensive evaluation of classifier performance independent of specific threshold selection by integrating precision-recall trade-offs across all possible decision boundaries. Formally:

$$\mathrm{AUC}=\int_{0}^{1}p\left(r\right)dr$$

where *p* denotes precision and *r* recall. PR-AUC is particularly sensitive to performance in positive classes, making it ideal for evaluating medical detection systems where lesion identification (positive class) is critical despite class imbalance.

## 3. Results

### 3.1. Dataset

To evaluate the performance of deep learning models for the analysis of bone tumors in radiographs, we utilized two datasets: the Bone Tumor X-ray Radiograph Dataset (BTXRD) and a retrospective dataset from the Interdisciplinary Musculoskeletal Tumor Center of the Technical University of Munich (referred to as the Munich dataset).

#### 3.1.1. BTXRD

BTXRD is a publicly available bone tumor radiograph dataset developed to address the lack of large, labeled datasets for primary bone tumor research [5]. It contains a total of 3746 X-ray images collected from three sources: (1) three hospitals in China, namely the First Affiliated Hospital of Guangxi Medical University, the Affiliated Hospital of Youjiang Medical College for Nationalities, and Baise People’s Hospital; (2) the Radiopaedia.org platform; and (3) the MedPix database. The dataset includes 1879 normal bone images, 1525 benign tumor images, and 342 malignant tumor images. Each image is annotated with metadata (gender, age, anatomical site, and view angle), and tumor images are labeled with diagnosis (benign/malignant), subtype, and anatomical site. The dataset comprises a total of nine bone tumor types, including osteochondroma, osteosarcoma, multiple osteochondromas, giant cell tumor, and others.

#### 3.1.2. Munich Dataset

This retrospective dataset consists of 934 radiographs from 934 patients collected between January 2000 and June 2020 [14]. All cases were histopathologically confirmed and included benign tumors (e.g., osteochondroma, enchondroma, chondroblastoma) and malignant tumors (e.g., osteosarcoma, Ewing sarcoma, lymphoma). Radiographs were reviewed independently by two radiologists in a blinded fashion. Patients with poor image quality were excluded. The dataset was split into training (70%), validation (15%), and testing (15%) subsets in a stratified manner. An additional external test set of 111 radiographs from Freiburg University Hospital was used for geographic validation.

#### 3.1.3. Dataset Statistics

Table 1 summarizes the distribution of samples across categories for both datasets.

Both datasets were annotated by multiple radiologists, ensuring that there was no potential inter-rater variability in annotations.

### 3.2. Comparison with SOTA Methods

To comprehensively evaluate the performance of our proposed YOLOv11-MTB method on both segmentation and classification tasks, we designed cross-dataset and cross-center comparison experiments. For the detection task, we selected state-of-the-art (SOTA) models including Mask R-CNN [24], YOLOv5 [22], YOLOv7 [25], and YOLOv8 [26] as baseline methods. For the classification task, we compared with CLIP [27], ViT [28], Swin [29], and BLIP [30].

In the classification task, we designed cross-dataset and cross-center experiments to assess the generalization and accuracy of each method. For the BTXRD dataset, we used data from Center 1 and Center 2 as the training set and data from Center 3 as the test set. For the Munich dataset, we used internal data as the training set and external data as the test set. To further validate model generalization, we also conducted cross-dataset experiments using the entire BTXRD dataset and the entire Munich dataset as training and test sets, respectively, or vice versa.

The detailed experimental results are presented in Table 2. In all experimental settings, our model demonstrates outstanding performance, particularly in cross-center and cross-dataset validations, indicating strong generalization capabilities. Significant accuracy improvements over SOTA baselines were validated by independent t-tests across all experimental configurations (all p<0.01). All models utilized pre-trained weights, except for the ViT model, which employed a full model training strategy. The other models used a frozen backbone with fine-tuning of the classification head. All models were trained with the same hyperparameter settings, including learning rate, batch size, and number of epochs, to ensure a fair comparison.

From the experimental results, it can be observed that while the performance differences among models are relatively small in the internal classification tasks of individual datasets, the YOLOv11-MTB model excels in cross-dataset and cross-center validations. In contrast, the other models, particularly CLIP and ViT, show significant performance drops in these scenarios, with notable decreases in accuracy and F1-score. This indicates that the YOLOv11-MTB model possesses stronger robustness and generalization capabilities for bone tumor classification tasks across different datasets and centers.

**Figure 3:**
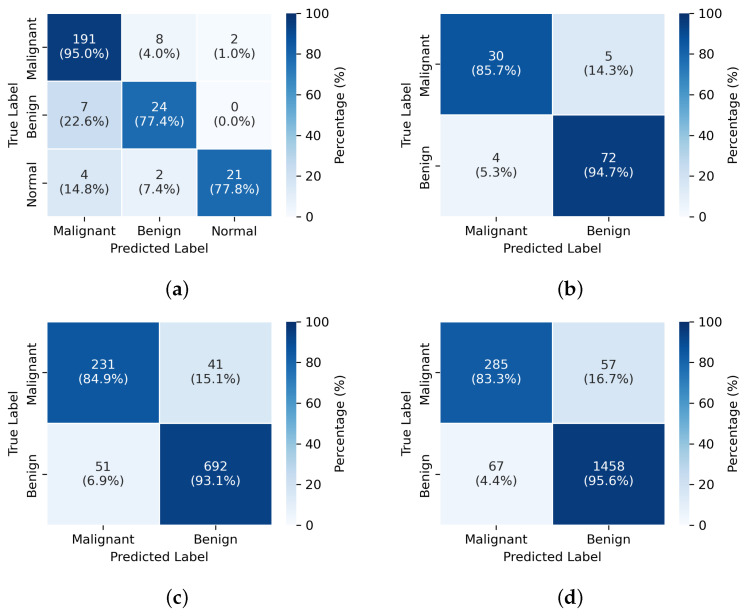
Confusion Matrices Under Four Experimental Configurations. The experimental setup for (**a**–**d**) is consistent with the order of Exp1–4 in Table 3.

In the detection task, we also employed cross-dataset and cross-center experimental designs to evaluate model performance. All models used pre-trained weights and were trained under the same hyperparameter settings. We selected Mask R-CNN, YOLOv5, YOLOv7, and YOLOv8 as baseline methods and compared them with our YOLOv11-MTB model. All models were trained and tested on the BTXRD dataset, using the same evaluation metrics, including mAP, F1-score, and FPS.

The results of the detection task are presented in Table 4. Our YOLOv11-MTB model consistently outperforms the baseline methods across all experimental settings, particularly in cross-dataset and cross-center validations. The mAP for small lesions and multi-lesion scenarios shows significant improvements, highlighting the effectiveness of our architectural enhancements. The F1-score also indicates superior performance in detecting small and multi lesions, which are critical for accurate tumor diagnosis.

**Figure 4:**
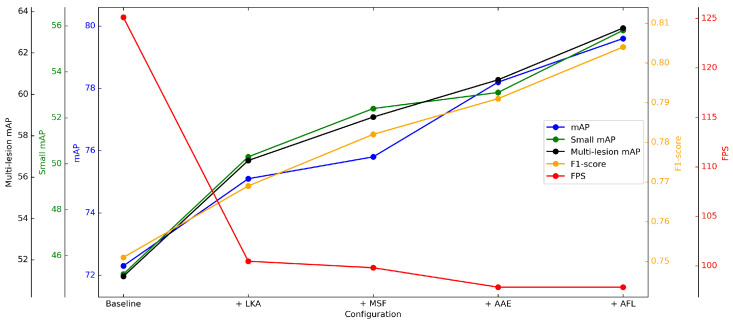
Component Ablation Analysis: Evolution of Key Performance Metrics.

### 3.3. Ablation Study

To evaluate the contributions of each architectural enhancement, we conducted a series of ablation studies on the BTXRD dataset. Each experiment was designed to isolate the impact of specific modifications on model performance, using the same training and testing protocols. Table 3 summarizes the results.

The components are chosen for their specific advantages in addressing the challenges in bone fracture detection: large kernel attention enhances the receptive field for better capturing context around small fractures, multi-scale fusion improves the detection of lesions at various sizes, angle-aware encoding handles the diverse orientations of fractures, and adaptive focal loss addresses the class imbalance.

Incorporating the large kernel attention (LKA) module resulted in an increase of overall mAP from 72.3% to 75.1%, with a notable improvement of 5.1% in small lesion detection. This demonstrates the benefit of capturing larger contextual information, which is particularly important in radiographic images with blurred or ambiguous lesion boundaries.

The multi-scale fusion module pushes the score to 75.8%. This indicates the importance of integrating multi-resolution features for accurate localization of lesions across different sizes.

Next, the introduction of angle-aware encoding led to further improvements, particularly in multi-lesion scenarios, where the mAP increased from 58.9% to 60.7%, while the overall mAP gain was moderate (+2.4%), the targeted benefit for complex cases validates the utility of view-specific spatial encoding in leveraging anatomical priors.

Finally, the integration of adaptive focal loss achieved the best overall performance, boosting the mAP to 79.6%, with the small lesion and multi-lesion mAP reaching 55.8% and 63.2%, respectively. This loss function effectively mitigates class imbalance and improves sensitivity to rare or difficult cases, which is crucial in clinical diagnosis.

Although these enhancements introduced a decrease in inference speed (from 125.10 FPS to 97.85 FPS), the final model maintains real-time processing capability, ensuring practical applicability in clinical environments.

### 3.4. Visualization

**Figure 5:**
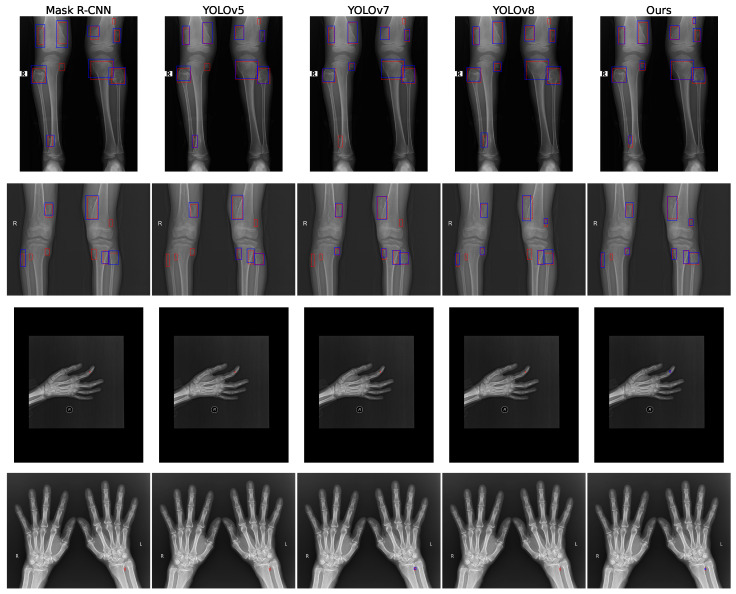
Visualization of detection results from the YOLOv11-MTB and other models on test images. Red dashed boxes indicate ground truth annotations, while blue dashed boxes show model detections. Only detections with IoU > 0.5 are displayed. The top two rows and bottom two rows show the prediction results of each model for typical images of multiple lesions and small lesions, respectively.

To further illustrate the effectiveness of our proposed YOLOv11-MTB model, we visualized the detection results on a sample of test images from the BTXRD dataset. Figure 5 shows the model’s ability to accurately localize and classify bone tumors, including small lesions and complex multi-lesion scenarios. The bounding boxes are color-coded based on the detected class, with confidence scores displayed. The red dashed boxes represent the ground truth annotations, while the blue dashed boxes indicate the model’s detection results. Only detections with an intersection over union (IoU) greater than 0.5 with the ground truth are shown, and the results have been processed using non-maximum suppression (NMS) to eliminate redundant detections.

From Figure 5, we observe that our method exhibits a low false negative rate, successfully detecting small lesions that other methods miss. For instance, in the first row, the small lesion in the upper right corner is detected only by our model. In the third row, while other methods fail to detect small lesions, our model successfully identifies them. The fourth row shows similar results, where only YOLOv7 and our method detect the target. Overall, our approach demonstrates superior performance in both small and multi-lesion detection tasks, attributed to the multi-scale feature fusion and large kernel attention modules we introduced, which enhance the model’s ability to capture critical information in complex scenarios.

## 4. Discussion

### 4.1. Clinical Significance and Performance Analysis

The results of this study demonstrate that the proposed YOLOv11-MTB framework achieves significant improvements in bone tumor detection, particularly for clinically challenging scenarios involving small lesions and multiple tumor sites. With an overall mAP of 79.6% and small-lesion mAP of 55.8%, our method addresses critical limitations in current computer-aided diagnostic systems for musculoskeletal oncology.

The substantial improvement in small lesion detection (55.8% vs. 45.2% baseline) is particularly relevant from a clinical perspective, as early-stage bone tumors often present as subtle radiographic abnormalities that may be overlooked during routine screening. The ability to detect lesions smaller than 32 × 32 pixels could potentially lead to earlier diagnosis and improved patient outcomes, especially for aggressive malignancies such as osteosarcoma where early intervention is crucial for limb salvage and survival [34].

Similarly, the enhanced performance in multi-lesion scenarios (63.2% mAP) addresses the clinical reality of metastatic bone disease and multifocal primary tumors, which present complex diagnostic challenges. The angle-aware encoding mechanism proves particularly valuable in this context, as it enables the model to leverage anatomical priors specific to different radiographic projections, reducing view-dependent false positives that commonly plague automated detection systems. However, it is important to note that while the model excels in detecting small and multiple lesions, there is a potential concern regarding the burden of false positives in clinical deployment. Radiologists may face an increased workload due to false alarms, which could lead to unnecessary follow-up imaging and delays in diagnosis. Therefore, future work should focus on minimizing false positives and improving the model’s precision in clinical settings.

### 4.2. Architectural Innovations and Technical Contributions

The integration of large kernel attention (LKA) modules represents a key architectural innovation that directly addresses the unique challenges of radiographic image analysis. Unlike natural images, X-ray radiographs exhibit complex overlapping anatomical structures and subtle contrast variations that require enhanced contextual understanding. The LKA mechanism with kernel size 31 and dilation factor 2 effectively captures long-range spatial dependencies while maintaining computational efficiency, as evidenced by the 2.8% improvement in overall mAP with only a modest increase in parameters.

The hierarchical multi-scale feature fusion strategy, inspired by the BiFPN architecture, demonstrates particular effectiveness for small object detection. The learnable weighted fusion mechanism allows the network to dynamically prioritize informative features across different scales, which is crucial given the variable size and morphology of bone tumors. This adaptive approach contrasts with fixed-weight fusion strategies commonly used in medical imaging and contributes to the observed improvements in detection sensitivity.

The adaptive focal loss implementation addresses a critical challenge in medical AI: severe class imbalance between normal, benign, and malignant cases. By dynamically adjusting the focusing parameter based on lesion characteristics (γ=2.5 for small lesions vs. γ=2.0 for larger lesions), the loss function effectively emphasizes hard-to-detect instances while maintaining overall classification accuracy. This targeted approach to class balancing represents a significant advancement over standard focal loss implementations in medical imaging applications.

### 4.3. Generalization and Robustness Analysis

The cross-dataset and cross-center validation experiments provide strong evidence for the generalizability of our approach. The relatively stable performance across different imaging centers and patient populations (mAP ranging from 77.4% to 79.6% across experiments) suggests that the learned features are robust to variations in imaging protocols, patient demographics, and institutional practices. This generalizability is crucial for clinical deployment, as medical AI systems must perform reliably across diverse healthcare settings.

The cross-dataset experiments (BTXRD → Munich and vice versa) are particularly informative, as they simulate real-world deployment scenarios where models trained at one institution are applied at another. The maintained performance levels (87.62% and 88.74% accuracy in classification tasks) indicate that our architectural enhancements successfully capture generalizable radiographic features rather than dataset-specific artifacts.

However, the performance variations observed across different experimental settings highlight the ongoing challenge of domain adaptation in medical imaging, while our model demonstrates superior robustness compared to baseline methods, there remains room for improvement in cross-institutional generalization, particularly for rare tumor subtypes that may be underrepresented in the training data.

### 4.4. Computational Efficiency and Clinical Deployment Considerations

The modest reduction in inference speed (from 89.2 to 80.9 FPS) represents an acceptable trade-off for the substantial performance gains achieved. The final processing rate of approximately 81 FPS remains well within real-time requirements for clinical applications, enabling integration into existing radiology workflows without significant computational bottlenecks.

The parameter increase from baseline YOLOv11 (approximately 15.4 M parameters) positions our model as computationally efficient compared to larger Transformer-based architectures while maintaining state-of-the-art performance. This efficiency is particularly important for deployment in resource-constrained clinical environments where computational resources may be limited.

From a workflow integration perspective, the object detection paradigm aligns well with radiologist interpretation patterns, providing localized predictions with confidence scores that can serve as decision support rather than replacement for clinical expertise. The bounding box outputs can be readily integrated into picture archiving and communication systems (PACS) for streamlined review and reporting.

### 4.5. Limitations and Future Directions

Several limitations of this study warrant consideration. First, while this study was validated on multi-center retrospective datasets, which enhances the model’s generalizability to some extent, prospective validation using unseen clinical data is essential to confirm its real-world applicability and is a necessary step before clinical trials. Additionally, the current datasets remain relatively limited in terms of tumor subtype diversity and variations in imaging protocols. Future work should incorporate larger, more diverse datasets with comprehensive histopathological correlation to further holistically validate its clinical utility.

Second, the binary classification approach (benign vs. malignant) represents a simplification of the complex diagnostic decision-making process in musculoskeletal oncology. Real-world applications would benefit from multi-class classification systems that can distinguish between specific tumor types and provide more granular diagnostic information.

Third, the current framework operates on individual 2D radiographs without leveraging temporal information from follow-up imaging or integration with clinical metadata such as patient age, symptoms, and laboratory findings. Incorporating such multimodal information could significantly enhance diagnostic accuracy and clinical relevance.

Fourth, external validation on prospective unseen data is essential for assessing the real-world clinical applicability of the model, while this study demonstrates strong performance across datasets, it is critical to validate the model on data that was not part of the training or retrospective validation process. This external validation will be crucial for the successful deployment of this system in clinical trials.

Another limitation is that the model currently focuses on image-level annotations rather than patient-level predictions. This distinction is crucial in clinical practice, where diagnosis and treatment decisions are often made at the patient level, accounting for the whole clinical context, including multiple lesions and their respective locations within the same patient. Future work should explore incorporating patient-level predictions, which would require integrating information from multiple images per patient to improve overall diagnostic accuracy.

The absence of uncertainty quantification mechanisms represents another area for improvement. Clinical deployment requires not only accurate predictions but also reliable confidence estimates to guide appropriate use of automated recommendations. Future iterations should incorporate uncertainty estimation techniques to provide calibrated confidence scores.

### 4.6. Broader Implications for Medical AI

This work contributes to the growing body of evidence supporting the clinical utility of deep learning in medical imaging, while highlighting the importance of domain-specific architectural adaptations. The success of our targeted modifications (LKA, multi-scale fusion, angle-aware encoding) demonstrates that generic computer vision architectures require careful adaptation for medical applications.

The emphasis on small object detection and multi-lesion scenarios addresses clinically relevant challenges that are often overlooked in general-purpose detection systems. This focus on clinically meaningful metrics, rather than purely technical benchmarks, represents an important shift toward more clinically oriented AI development.

The demonstrated generalizability across multiple centers and datasets provides encouraging evidence for the potential real-world deployment of such systems. However, the path to clinical implementation requires continued validation, regulatory approval, and careful integration with existing clinical workflows to ensure patient safety and diagnostic accuracy.

Future research should focus on developing comprehensive multimodal systems that integrate radiographic analysis with clinical data, implementing robust uncertainty quantification, and conducting prospective clinical trials to establish real-world efficacy and safety profiles. The foundation established by this work provides a promising starting point for such translational research efforts.

## 5. Conclusions

This research introduces a comprehensively enhanced YOLOv11 architecture specifically optimized for bone tumor detection in X-ray images. Our approach systematically addresses critical challenges in musculoskeletal oncology imaging through four key innovations: large kernel attention modules that capture long-range contextual relationships in complex anatomical backgrounds, multi-scale feature fusion with learnable weighting to improve small lesion detection, angle-aware positional encoding for viewpoint invariance across diverse imaging projections, and adaptive class re-weighting to mitigate substantial data imbalance. Validation across multi-center datasets demonstrates significant performance improvements over baseline models, with particularly notable gains in clinically critical areas such as early-stage malignancy identification and multi-lesion detection.

The proposed framework achieves state-of-the-art performance in both detection and classification tasks, showing substantial advances in detecting sub-centimeter lesions and discriminating malignant tumors. The architecture’s robustness across varying imaging angles and multi-lesion scenarios positions it as a promising computer-aided diagnostic tool with significant clinical translation potential. These technical advancements directly address the pressing need for reliable detection systems in orthopedic oncology practice. Future research will focus on prospective clinical validation to assess real-world applicability and the development of an open-source software tool for seamless integration into diagnostic workflows.

## Figures and Tables

**Table 1:** Statistics of the BTXRD and Munich datasets.

Dataset	Normal	Benign Tumor	Malignant Tumor	Total
BTXRD (Center 1)	1593	1110	235	2938
BTXRD (Center 2)	259	214	76	549
BTXRD (Center 3)	27	201	31	259
BTXRD Total	1879	1525	342	3746
Munich (Internal)	–	667	267	934
Munich (External)	–	76	35	111

**Table 2:** Cross-dataset and cross-center performance comparison for tumor classification.

Experiment	Model	Accuracy	Precision	Recall	F1-Score	AUC
Exp1: BTXRD Center1/2 → Center3	CLIP [31]	83.21 ± 0.83	81.35 ± 0.75	79.42 ± 0.93	80.36 ± 0.85	88.15 ± 0.68
	ViT [32]	85.73 ± 0.61	83.97 ± 0.53	82.15 ± 0.75	83.04 ± 0.65	89.67 ± 0.58
	Swin [29]	87.92 ± 0.54	85.63 ± 0.45	84.27 ± 0.63	84.93 ± 0.58	91.24 ± 0.40
	BLIP [33]	86.45 ± 0.72	84.12 ± 0.69	83.56 ± 0.81	83.83 ± 0.73	90.17 ± 0.51
	Ours	91.38 ± 0.49	89.76 ± 0.33	88.93 ± 0.51	89.34 ± 0.42	94.62 ± 0.39
Exp2: Munich Internal → External	CLIP	82.16 ± 1.21	78.34 ± 1.36	80.25 ± 1.13	79.28 ± 1.29	87.43 ± 0.84
	ViT	84.95 ± 0.96	81.67 ± 1.02	83.14 ± 0.85	82.39 ± 0.92	89.26 ± 0.73
	Swin	86.32 ± 0.82	83.45 ± 0.90	84.97 ± 0.79	84.20 ± 0.84	90.85 ± 0.64
	BLIP	85.14 ± 1.02	82.78 ± 1.14	83.56 ± 0.95	83.16 ± 1.02	89.73 ± 0.73
	Ours	89.87 ± 0.72	87.62 ± 0.89	88.34 ± 0.66	87.98 ± 0.78	93.45 ± 0.53
Exp3: BTXRD → Munich	CLIP	70.54 ± 1.87	68.12 ± 1.92	69.23 ± 1.85	68.67 ± 1.89	84.12 ± 1.76
	ViT	61.67 ± 7.15	59.12 ± 6.89	60.45 ± 7.32	59.78 ± 7.10	74.12 ± 6.45
	Swin	75.12 ± 2.32	73.45 ± 2.12	74.12 ± 2.45	73.78 ± 2.32	82.45 ± 2.12
	BLIP	80.26 ± 0.84	78.14 ± 0.91	79.27 ± 0.70	78.70 ± 0.83	86.87 ± 0.61
	Ours	87.62 ± 0.81	85.93 ± 0.60	86.45 ± 0.65	86.19 ± 0.58	92.78 ± 0.47
Exp4: Munich → BTXRD	CLIP	75.93 ± 1.12	73.45 ± 1.25	74.12 ± 1.15	73.78 ± 1.18	82.45 ± 1.05
	ViT	57.45 ± 6.78	55.12 ± 6.45	56.34 ± 6.89	55.78 ± 6.67	70.12 ± 5.98
	Swin	85.93 ± 0.57	83.25 ± 0.64	84.76 ± 0.42	84.00 ± 0.50	90.62 ± 0.33
	BLIP	82.47 ± 0.61	79.83 ± 0.78	81.25 ± 0.54	80.53 ± 0.69	87.95 ± 0.42
	Ours	88.74 ± 0.41	86.82 ± 0.59	87.63 ± 0.31	87.22 ± 0.49	93.87 ± 0.31

Note: All metrics are in percentage (%). Exp1 uses three-class macro averaging while others are binary classification. Detailed confusion matrix of our method. See Figure 3.

**Table 3:** Ablation study results on BTXRD dataset showing the contribution of each component.

Configuration	mAP	Small mAP	Multi-Lesion mAP	F1-Score	FPS
Baseline YOLOv11	72.3	45.2	51.2	0.751	125.10
+ Large Kernel Attention	75.1	50.3	56.8	0.769	100.47
+ Multi-scale Fusion	75.8	52.4	58.9	0.782	99.81
+ Angle-aware Encoding	78.2	53.1	60.7	0.791	97.85
+ Adaptive Focal Loss	79.6	55.8	63.2	0.804	97.85

Note: FPS evaluated using YOLOv11’s native acceleration (e.g., FP16 inference). See Figure 4 for visualization results.

**Table 4:** Cross-dataset and cross-center performance comparison for tumor detection.

Experiment	Model	mAP	Small mAP	Multi-Lesion mAP	F1-Score	FPS	Params (M)
Exp1: BTXRD Center1/2 → Center3	Mask R-CNN	65.8 ± 1.12	33.2 ± 1.25	38.5 ± 1.15	0.682 ± 1.08	95.4 ± 1.3	42.3
	YOLOv5	69.6 ± 0.92	38.5 ± 1.05	42.8 ± 0.95	0.718 ± 0.88	120.3 ± 1.2	7.2
	YOLOv7	71.2 ± 0.83	40.8 ± 0.95	45.2 ± 0.88	0.735 ± 0.82	115.6 ± 1.1	9.6
	YOLOv8	73.1 ± 0.75	43.2 ± 0.88	48.5 ± 0.82	0.752 ± 0.78	110.4 ± 1.3	10.9
	Ours	79.6 ± 0.62	55.8 ± 0.71	63.2 ± 0.65	0.804 ± 0.68	80.9 ± 1.5	15.4
Exp2: Munich Internal → External	Mask R-CNN	67.3 ± 1.22	35.5 ± 1.35	40.8 ± 1.22	0.692 ± 1.25	92.8 ± 1.4	42.3
	YOLOv5	67.6 ± 1.18	36.2 ± 1.32	41.5 ± 1.15	0.698 ± 1.25	118.7 ± 1.4	7.2
	YOLOv7	69.0 ± 1.12	37.8 ± 1.25	42.7 ± 1.05	0.712 ± 1.18	116.5 ± 1.3	9.6
	YOLOv8	71.2 ± 0.98	40.5 ± 1.12	45.8 ± 0.95	0.735 ± 1.05	113.2 ± 1.1	10.9
	Ours	78.3 ± 0.81	50.2 ± 0.92	58.1 ± 0.76	0.792 ± 0.85	81.4 ± 1.4	15.4
Exp3: BTXRD → Munich	Mask R-CNN	62.5 ± 1.42	31.8 ± 1.52	35.4 ± 1.35	0.658 ± 1.35	92.8 ± 1.6	42.3
	YOLOv5	65.8 ± 1.12	34.8 ± 1.25	39.2 ± 1.05	0.685 ± 1.18	117.9 ± 1.4	7.2
	YOLOv7	67.9 ± 0.98	37.5 ± 1.12	42.0 ± 0.95	0.705 ± 1.05	114.8 ± 1.3	9.6
	YOLOv8	70.1 ± 0.88	40.2 ± 0.98	45.5 ± 0.83	0.725 ± 0.92	111.5 ± 1.2	10.9
	Ours	77.4 ± 0.72	51.3 ± 0.81	59.4 ± 0.65	0.780 ± 0.78	79.6 ± 1.5	15.4
Exp4: Munich → BTXRD	Mask R-CNN	63.8 ± 1.32	32.5 ± 1.42	36.5 ± 1.25	0.670 ± 1.28	91.9 ± 1.5	42.3
	YOLOv5	66.3 ± 1.15	35.2 ± 1.28	39.7 ± 1.12	0.690 ± 1.22	116.2 ± 1.3	7.2
	YOLOv7	68.5 ± 1.02	37.8 ± 1.15	42.3 ± 0.98	0.710 ± 1.08	113.4 ± 1.2	9.6
	YOLOv8	70.8 ± 0.91	40.3 ± 1.05	45.8 ± 0.88	0.730 ± 1.02	110.1 ± 1.4	10.9
	Ours	78.0 ± 0.75	52.4 ± 0.85	60.8 ± 0.68	0.790 ± 0.82	80.3 ± 1.6	15.4

Note: All metrics are in percentage (%) except for FPS and Params. Small mAP refers to mAP for lesions <32 pixels, and Multi-lesion mAP refers to mAP for images with 3+ lesions. FPS reported without inference acceleration techniques.

## Data Availability

The BTXRD dataset was obtained from the Bone Tumor X-ray Radiograph Dataset (BTXRD), which is publicly available at https://www.kaggle.com/datasets/thanhngan123/btxrd-data/data (accessed on 1 March 2025). The Munich dataset was obtained from the Interdisciplinary Musculoskeletal Tumor Center of the Technical University of Munich, which is available on request from the corresponding author of [14] due to the privacy of data.
